# Early Local Inhibition of Club Cell Protein 16 Following Chest Trauma Reduces Late Sepsis-Induced Acute Lung Injury

**DOI:** 10.3390/jcm8060896

**Published:** 2019-06-22

**Authors:** Philipp Störmann, Nils Becker, Jan Tilmann Vollrath, Kernt Köhler, Andrea Janicova, Sebastian Wutzler, Frank Hildebrand, Ingo Marzi, Borna Relja

**Affiliations:** 1 Department of Trauma, Hand and Reconstructive Surgery, University Hospital Frankfurt, 60590 Frankfurt, Germany; Philipp.Stoermann@kgu.de (P.S.); N.Becker2@gmx.net (N.B.); Tilmann.Vollrath@kgu.de (J.T.V.); andrea.janicova@gmail.com (A.J.); Ingo.Marzi@kgu.de (I.M.); 2 Institute of Veterinary Pathology, Justus Liebig University Giessen, 35392 Giessen, Germany; kernt.koehler@vetmed.uni-giessen.de; 3 Orthopedic and Trauma Surgery, Helios Horst Schmidt Kliniken, 65199 Wiesbaden, Germany; swutzler@yahoo.de; 4 Department of Trauma Surgery, Hospital of the RWTH University, 52074 Aachen, Germany; fhildebrand@ukaachen.de

**Keywords:** uteroglobin, CC16, inflammation, chest trauma, acute lung injury (ALI)

## Abstract

Blunt thoracic trauma (TxT) deteriorates clinical post-injury outcomes. Ongoing inflammatory changes promote the development of post-traumatic complications, frequently causing Acute Lung Injury (ALI). Club Cell Protein (CC)16, a pulmonary anti-inflammatory protein, correlates with lung damage following TxT. Whether CC16-neutralization influences the inflammatory course during ALI is elusive. Ninety-six male CL57BL/6N mice underwent a double hit model of TxT and cecal ligation puncture (CLP, 24 h post-TxT). Shams underwent surgical procedures. CC16 was neutralized by the intratracheal application of an anti-CC16-antibody, either after TxT (early) or following CLP (late). Euthanasia was performed at 6 or 24 h post-CLP. Systemic and pulmonary levels of IL-6, IL-1β, and CXCL5 were determined, the neutrophils were quantified in the bronchoalveolar lavage fluid, and histomorphological lung damage was assessed. ALI induced a significant systemic IL-6 increase among all groups, while the local inflammatory response was most prominent after 24 h in the double-hit groups as compared to the shams. Significantly increased neutrophilic infiltration upon double hit was paralleled with the enhanced lung damage in all groups as compared to the sham, after 6 and 24 h. Neutralization of CC16 did not change the systemic inflammation. However, early CC16-neutralization increased the neutrophilic infiltration and lung injury at 6 h post-CLP, while 24 h later, the lung injury was reduced. Late CC16-neutralization increased neutrophilic infiltration, 24 h post-CLP, and was concurrent with an enhanced lung injury. The data confirmed the anti-inflammatory potential of endogenous CC16 in the murine double-hit model of ALI.

## 1. Introduction

Chest (thoracic) trauma (TxT) is one of the most common injuries presented to emergency departments, and it constitutes the third most common cause of death in severely injured patients [1,2]. Severe lung contusions occur in every fourth multiple-injury trauma patients [3,4]. It is well-known that next to the primary impact of chest trauma itself, ongoing, post-traumatic, systemic, and inflammatory changes caused by secondary stimuli (e.g., surgeries or infections) contribute to the development of severe pulmonary complications, including Acute Lung Injury (ALI) and its most severe form, Acute Respiratory Distress Syndrome (ARDS) [5,6].

With regard to this “two-hit” hypothesis, it has been widely demonstrated in animal models that isolated experimental lung damage was inappropriate to simulate the complex immune mechanisms causing Acute Lung Injury (ALI) [7]. Although isolated blunt chest trauma in mice induced a profound inflammatory reaction, it was not sufficient to establish ongoing pathological pulmonary changes, due to the fast recovery within 24 hours after trauma [8]. Considering the variety of established animal models, the use of cecal ligation and puncture (CLP) as a second hit was demonstrated, to a reasonable extent, to induce and mimic the human etiology of indirect lung damage ending in ALI [8]. Thus, a combination of different models is necessary to induce the mechanisms leading to ALI, and such a combinatory model addressing the double-hit hypothesis might overcome the failings of single-hit models [7]. Combining blunt chest trauma with a secondary CLP induces lung apoptosis, capillary leakage, and alterations in lung histology, which are typical for ALI development [8,9,10]. However, the exact mechanisms of ALI development still remain elusive. It is still not evident if ALI upon blunt chest trauma is mainly driven by either direct local tissue injury and its related inflammatory changes, or if it is rather driven by the second hit, in this case CLP, which itself causes a strong systemic inflammatory reaction, leading to the so-called remote organ damage [8,11].

Recently, we have demonstrated that the earlier observed lung injury with moderate inflammatory changes after blunt chest trauma recovered quickly, and therefore, might have been caused by mechanical lung injury [8]. In contrast, lung injury followed by CLP as a second hit, did not recover and was concurrent with significant inflammation [8]. In addition to the well-described pro-inflammatory mediators characterizing the acute inflammatory process in the lung tissue and airways, which are known to induce an acute onset of severe oxygenation disturbances due to the loss of barrier function of the lung epithelial and capillary endothelial cells, endogenous anti-inflammatory factors were also shown to play an important role in pulmonary complications [8,12].

Club Cell protein (CC)16 was described as an anti-inflammatory protein that is derived from lung epithelial club cells [13,14,15]. The systemic concentrations of this anti-inflammatory, lung-specific protein, correlated with the extent of pulmonary contusion and chest complications in traumatized patients, indicating its biomarker characteristics [16,17,18,19]. The anti-inflammatory biology of CC16 has been confirmed as being protective in the development of chronic obstructive pulmonary disease (COPD) [14,20]. Interestingly, increased CC16 levels in patients with Idiopathic Pulmonary Fibrosis (IPF) suggest that it might participate in disease pathogenesis [13]. CC16 was not only found in club cells but also in alveolar epithelial cells [13]. Thus, aside from its proposed clinical biomarker characteristic to predict the occurrence of respiratory complications, there is accumulating evidence that CC16 might exert important pathophysiological effects during the development of lung complications. It is well-established that an inflammatory response after trauma is essential for host defense, but that it can cause further tissue damage if triggered by a secondary stimulus [21,22]. Reducing the inflammation attenuated the pathological injury and survival upon ALI, in mice [23]. On the other hand, immunoparalysis, which has been closely associated with posttraumatic infectious complications, is caused by an anti-inflammatory status of the organism, and CC16 as a strong local anti-inflammatory factor might play a pathological role here [24,25,26]. Therefore, we evaluated whether local neutralization of the anti-inflammatory CC16 influences the pulmonary inflammation after sepsis-induced ALI, following blunt chest trauma in a murine model. We hypothesized that via early neutralization of CC16, the early immunosuppression during CLP-induced ALI after blunt chest trauma will be abrogated, and this will be beneficial in the underlying model.

## 2. Experimental Section

### 2.1. Animals and Experimental Model

All experiments were conducted in accordance with the German Federal Law with regard to the protection of animals. This study was approved by the responsible government authority, the Veterinary Department of the Regional Council in Darmstadt, Germany (Regierungspräsidium Darmstadt, Hessen, Germany; AZ: FK 1068 from the 19 July 2016) and was performed in consent with the ARRIVE Guidelines [27].

Ninety-six male CL57BL/6N mice (25 ± 5 g) at 6–8 weeks of age were included (Janvier Labs, France). The sedation and analgesia were performed as described previously [8]. Before and after the experimental procedures, the animals had access to water and food ad libitum.

Mice underwent blunt chest trauma under general mask anesthesia, as described before [8,28]. Briefly, they were placed in a supine position under a centrally positioned cylinder 2.5 cm above the sternum, which was separated by a Mylar polyester film (0.05 mm, Du Pont, Bad Homburg, Germany). Blunt bilateral thoracic trauma (TxT) was induced by a standardized pressure wave provided directly to the chest.

**Figure 1:**
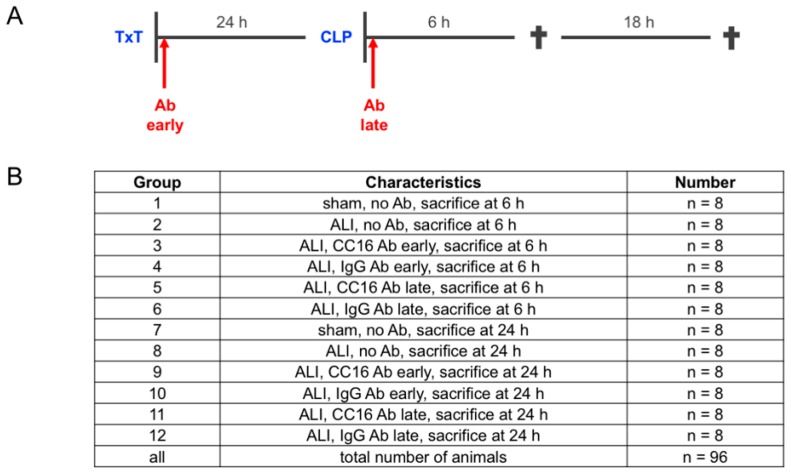
(**A**) Experimental design. Mice underwent the double hit consisting of thoracic trauma (TxT) and cecal ligation and puncture (CLP). The sham control group underwent identical surgical procedures and anesthesia, as described above, without performing TxT or CLP. Interventions with either CC16 antibody (Ab) or IgG control (IgG) antibody were performed either early (post TxT) or late (post-CLP) to the lungs. Six or 24 h later animals were euthanized for sampling. (**B**) Group allocation and group size is given.

Twenty-four hours later, the animals intraperitoneally received Ketamine (Zoetis, Berlin, Germany) and Xylazin (Bayer, Leverkusen, Germany). Then, a median laparotomy with moderate cecal ligation and puncture (CLP) was performed, as described before [8]. Briefly, the distant cecum was ligated, perforated using a 25 gauge cannula (Braun, Melsungen, Germany) and then placed in the abdominal cavity. The abdominal closure was performed using a two-layered suture. Eighty animals underwent the double-hit consisting of TxT and CLP, and were assigned to the ALI group. Sixteen animals in the sham control group underwent identical anesthesia procedures, as described above, without performing any surgical procedures. Either 6 or 24 h later, the animals were euthanized for sampling. The overview of the experimental design and group allocation is depicted in Figure 1.

### 2.2. Group Allocation According to the Application of Anti CC16-Antibody

Animals were randomly assigned to the experimental groups for either early (post-TxT) or late local antibody (Ab) application (post-CLP) to the lungs. Administration of the uteroglobin/SCGB1A1 (CC16 Ab, LS Biosciences, Seattle, WA, USA) or IgG Control (IgG) Antibody (10 µg/mL, R&D Systems, Minneapolis, MN, USA) was performed immediately after the induction of thoracic trauma, while the late group received antibodies, following the CLP (Figure 1A). For antibody administration, mice were placed in a supine position and the tongue was thoroughly kept aside. A buttoned cannula was placed at the beginning of the trachea, and 50 µL were carefully administered. Mice were then kept in a reverse Trendelenburg position, for 30 seconds, to ensure proper Ab distribution inside the lungs. Figure 1B shows all of the experimental groups.

### 2.3. Cytometric Bead Array for IL-6 and IL-10 Determination

The vena cava was punctured by a heparinized syringe for blood withdrawal, at 6 or 24 h after the CLP. Plasma was isolated from blood samples after centrifugation (1164× *g* for 15 min at 4 °C) and then stored at −80 °C, for cytokine measurement. Cytokines were measured with the BD CBA Mouse Inflammation Kit (BD Bioscience, San Jose, CA, USA). Interleukin-6 (IL-6) and IL-10 were determined in a single sample. The operations were performed according to the manufacturer’s instruction. Briefly, beads coated with specific capture antibodies were mixed, and 50 μL of this mixture, 50 μL of the plasma sample or standard dilutions, and 50 μL of the phycoerythrin (PE) detection reagent were added consecutively to each assay tube and incubated for 2 h, at room temperature, in the dark. Then, the samples were washed according to the manufacturer’s instruction and the bead pellet was resuspended in 200 μL buffer. Samples were measured using the BD FACS Canto II Flow Cytometer and were analyzed by the FCAP Array^TM^Software (BD Bioscience, San Jose, CA, USA).

### 2.4. Quantification of Protein Expression Levels in the Lungs

After blood withdrawal, the trachea was punctured and intubated. Then, the lungs were flushed with 1.2 mL phosphate buffered saline (PBS), to collect the bronchoalveolar lavage fluid (BAL) for later analyses. No selective lung BAL was performed. The animals were then perfused with 20 mL PBS via the caudal vena cava, and the lungs were subsequently removed. One lung lobe was snap-frozen using liquid nitrogen, and the other one was filled with 4% formalin, for an overnight fixation and subsequent (immuno)histological analyses. Lung tissue was homogenized in protein lysis buffer at 4 °C, followed by centrifugation for 30 min at 4 °C at 20,000× *g*. Supernatants were stored at −80 °C for later analysis. Protein concentrations of pulmonary CC16, IL-6, IL-1β, CXCL1, and CXCL5 were determined using a SCGB1A1 ELISA Kit (Secretoglobin, Family 1A, Member 1 (uteroglobin), antikoerper-online, Aachen, Germany) and Mouse IL-6, IL-1 beta/IL-1F2, CXCL1/KC, and LIX were measured using a DuoSet ELISA kit of R&D Systems, according to the manufacturer’s instructions (Wiesbaden-Nordenstadt, Germany). ELISA was performed using an Infinite M200 microplate reader (Tecan, Männedorf, Switzerland).

### 2.5. Detection of Granulocytes in the BAL by FLOW Cytometry

BAL samples were centrifuged at 1164× *g* at 4 °C for 5 min. The cell pellets were resuspended in 100 µL PBS, supplemented with 0.5% bovine serum albumin (FACS buffer), and 40 µL were transferred into polystyrene FACS tubes (BD, Heidelberg, Germany). Then, the samples were incubated with Pacific blue-conjugated anti-mouse Ly-6G antibody (Ab), APC/Fire 750 conjugated anti-mouse CD45 Ab, and phycoerythrin conjugated anti-mouse CD16/CD32 Ab (5 μL, BD Biosciences, Franklin Lakes, USA). Control staining with the corresponding isotype antibodies was applied to the setting. After 30 minutes on ice, 5 μL of 7-AAD (BD Biosciences, Franklin Lakes, USA) was added, and the samples were incubated for a further 15 minutes. Then, the samples were washed with 2 mL FACS buffer (7 min at room temperature (RT) and 423× *g*). The supernatants were removed and the cell pellet was homogenized in 1 mL BD FACS Lysing Solution, for an additional 10 min (RT). Then, the samples were centrifuged at 400× *g* for 7 min and washed twice with 2 mL FACS buffer. After removal of the supernatants, the cells were diluted in 80 µL FACS buffer and stored on ice until measurement. Each cell population was defined by gating the corresponding forward and side scatter scan as well as the viable cells, by applying the 7-AAD for gating. From each sample, a minimum of 3×10^4^ cells were measured, which were subsequently analyzed. The percentage of CD16^+^ out of Ly6G^+^CD45^+^ viable cells was assessed by flow cytometric analyses, using a BD FACS Canto 2™ and FACS DIVA^TM^ software (BD, Heidelberg, Germany).

### 2.6. Histological Examination of Lung Injury

After blood withdrawal and BAL sampling, the animals were perfused with 20 mL PBS via the caudal vena cava, and subsequently, the lungs were removed. One lung lobe was snap-frozen for CBA analyses, and the other one was filled with 4% formalin for overnight fixation. Thereafter, the specimen was embedded into the paraffin and sectioned into 2–3 µm sections and then prepared for subsequent staining with hematoxylin-eosin (HE). Determination of the histological tissue damage of HE-stained sections was performed by an independent examiner who allocated samples to the various experimental groups, in a blinded manner. Sections of the lungs were examined for desquamation, dystelectasis/atelectasis, emphysema, congestion, interstitial thickness/infiltration, and bronchial exudate, and were expressed as lung injury score (LIS). Five regions from each specimen were examined. Each parameter was assessed according to the degree of severity: 0 = not observed, 0.5 = minimal/discrete, 1 = mild, 2 = moderate, and 3 = marked. Then, the sum was calculated for a total score of lung injury. The parameters were chosen as previously published [29].

### 2.7. Staining of CC16

Paraffin-embedded lung samples were sectioned (3 µm), deparaffinized, rehydrated, and stained with the anti-CC16 antibody. After deparaffinization, epitope recovery was performed under a steam atmosphere, using R-Universal epitope recovery buffer (Aptum, Kassel, Germany) for one hour (Retriever 2100, Prestige Medical, Aptum, Southhsampton, UK). After washing with water and PBS, anti-uteroglobin antibody (Abcam, Cambridge, UK) was applied as a primary antibody. After the incubation for one hour at room temperature, and a subsequent washing procedure, a secondary AlexaFluor568 donkey anti-rabbit IgG antibody (1:100, Invitrogen, Carlsbad, California, US) was applied to detect specific binding. After another hour at room temperature, the sections were washed and mounted using the fluorescent mounting medium containing 4′,6-diamidino-2-phenylindole (DAPI) nucleic acid stain (Vectashield HardSet Antifade Mounting Medium with DAPI, Vector Laboratories Ltd, Cambridgeshire, UK). Fluorescence was visualized using a Zeiss inverted fluorescence microscope AXIO Observer Z1 (Carl Zeiss AG, Oberkochen, Germany).

### 2.8. Statistical Analyses

All analyses were performed using GraphPad Prism 6 (Graphpad Software Inc., SanDiego, CA, USA). Data were presented as box and whiskers (min to max). Based on the D’Agostino–Pearson normality test, the non-parametric Kruskal–Wallis test, which does not assume a normal distribution of the residuals, followed by Dunn’s post hoc test for the correction of multiple comparisons were applied. A *p* value of less than 0.05 was considered to be statistically significant.

## 3. Results

### 3.1. Study Data

#### 3.1.1. Presence of CC16 in Lung Tissue

**Figure 2:**
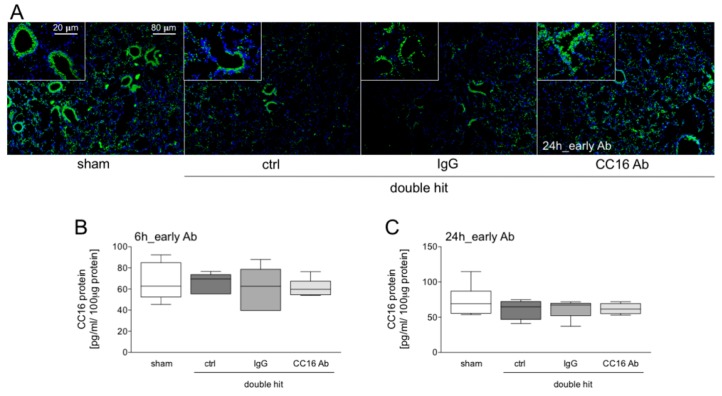
(**A**) CC16 (green) and nuclei (blue, 4′,6-diamidino-2-phenylindole, DAPI) staining 24 hours after CLP and early interventions with antibodies shows intrabronchial high concentrations of CC16 positive cells. In double-hit groups, a prominent loss of CC16 into the interstitium as well as a loss of the pulmonary integrity is shown. (**B**) Pulmonary CC16 levels are shown in animals with early interventions with either CC16 antibody (Ab) or IgG control (IgG) antibody, at 6 or 24 h post-CLP (**C**) (*n* = 8).

Figure 2 shows representative immunohistological stainings of CC16 upon early Ab administration and sampling after 24 hours. A prominent CC16 distribution in the terminal and respiratory bronchioles of the lung sections upon double hit is shown (Figure 2A). CC16 was released from the injured cells (Figure 2A). At 6 or 24 hours after CLP, CC16 levels were not significantly changed in all double-hit groups, compared to the corresponding sham groups, after 6 h as well as after 24 h (Figure 2B,C, respectively).

#### 3.1.2. IL-6 and IL-10 Concentrations in the Blood

**Figure 3:**
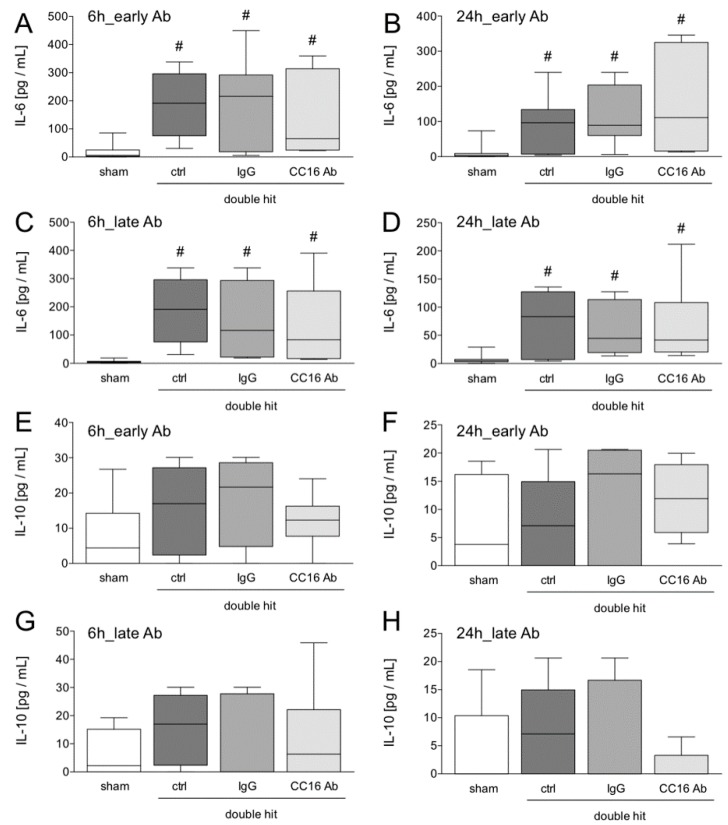
Systemic interleukin (IL)-6 (**A**–**D**) and IL-10 (**E**–**H**) levels are shown. Interventions with either the CC16 antibody (Ab) or IgG control (IgG) antibody were performed either early (post TxT, A–B and E–F) or late (post-CLP, C–D, and G–H) to the lungs. Six or 24 hours later, the animals were euthanized for sampling. #: *p* < 0.05 versus sham, *n* = 8.

The systemic inflammatory response induced by the double hit was detected at 6 or 24 hours after CLP (Figure 3). At both time points, IL-6 levels were significantly increased in all ALI groups, compared to the corresponding sham groups at 6 h, as well as at 24 h (*p* < 0.05, Figure 3A–D). No significant changes were detected between the groups with either early or late CC16 Ab or IgG Ab application as compared to the control groups, after the double-hit (Figure 3A–D).

Concentrations of circulating IL-10 were not significantly changed among the groups, although there was a trend of increased cytokine levels in all ALI groups, as compared to the shams (Figure 3E–H). Interestingly, a trend of decreased IL-10 at 24 hours after CLP and late CC16 Ab application was observed (Figure 3H).

#### 3.1.3. Quantification of Protein Expression Levels in the Lungs

**Figure 4:**
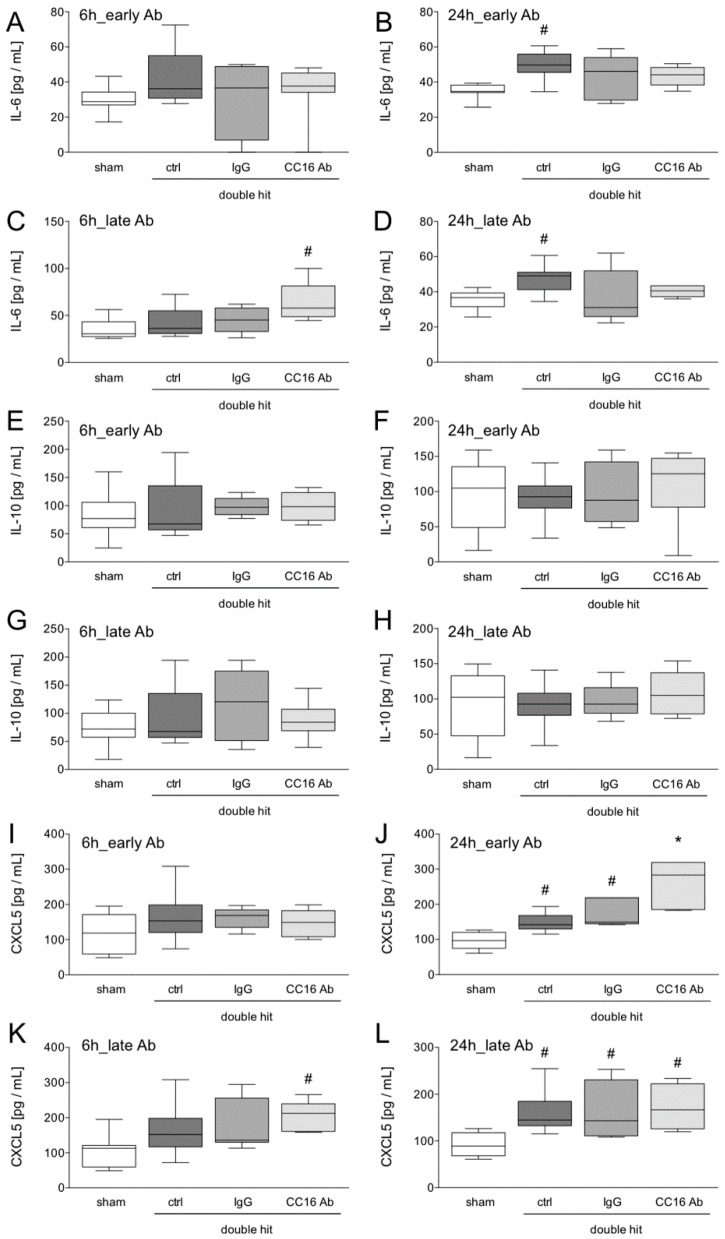
Pulmonary interleukin (IL)-6 (**A**–**D**), IL-10 (**E**–**H**) and CXCL5 (**I**–**L**) levels are shown. Interventions with either CC16 antibody (Ab) or IgG control (IgG) antibody were performed either early (post TxT, A–B, E–F, and I–J) or late (post-CLP, C–D, G–H, and K–L) to the lungs. Six or 24 hours later, the animals were euthanized for sampling. *: *p* < 0.05 versus all; #: *p* < 0.05 versus sham, *n* = 8.

At 6 hours after early Ab application no significant changes in IL-6 were observed (Figure 4A). The protein concentration of pulmonary IL-6 was significantly increased in the control group, 24 hours after the double hit (Figure 4B,D). The group with late CC16 Ab application after CLP had significantly increased protein levels of IL-6 in the lungs, compared to all other groups (*p* < 0.05, Figure 4C).

The local IL-10 concentrations did not significantly differ between the groups at any time point (Figure 4E–H).

While the group with early application of CC16 Ab had no significant changes in CXCR5 levels in the lungs, the group with the late CC16 Ab application, post-CLP, had significantly increased protein levels of CXCL5 in the lungs (*p* < 0.05, Figure 4K). Twenty-four hours after CLP, CXCL5 levels were significantly increased, compared to the shams (*p* < 0.05, Figure 4J,L). In the double-hit group that received the CC16 Ab early, significantly increased levels of CXCL5, compared to the sham and both other ALI groups (control and IgG) were detected (*p* < 0.05, Figure 4J).

#### 3.1.4. Detection of CD16^+^Ly6G^+^CD45^+^ Neutrophils in the BAL by Flow Cytometry

**Figure 5:**
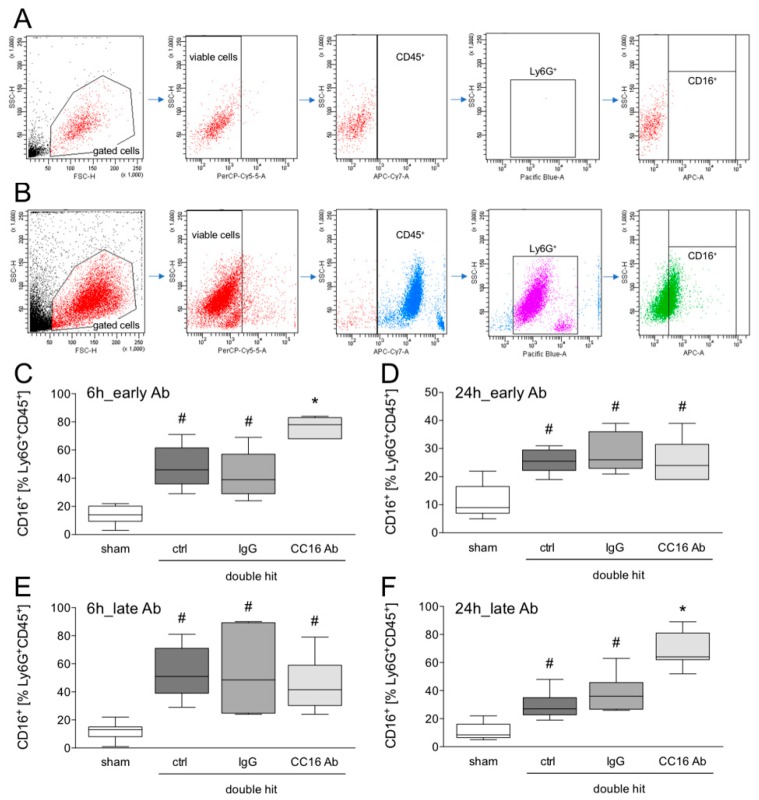
Flow cytometric analysis of CD16^+^ cells out of viable CD45^+^Ly6G^+^ cells in the bronchoalveolar fluid. Gating strategy with isotype control antibodies (**A**) and antibody stainings (**B**) is shown. Interventions with either CC16 antibody (Ab) or IgG control (IgG) antibody were performed, either early (post TxT, **C**–**D**) or late (post-CLP, **E**–**F**) to the lungs. Six or 24 hours later, the animals were euthanized for sampling. *: *p* < 0.05 versus all; #: *p* < 0.05 versus sham, *n* = 8.

To analyze whether and how CC16 modulates the neutrophil granulocyte immigration into the lungs after the double hit, the presence of CD16^+^Ly6G^+^CD45^+^ viable cells in the BAL was assessed. The gating strategy is shown in Figure 5A and B. ALI significantly increased the percentage of CD16^+^Ly6G^+^CD45^+^ cells in the control group, compared to the corresponding sham group at 6 and 24 hours after CLP (*p* <0.05, Figure 5C–F). This increase in CD16^+^Ly6G^+^CD45^+^ cells in the BAL was significant in the groups that received either the IgG Ab or the CC16 Ab, compared to the sham at 6 and 24 hours after CLP (*p* < 0.05, Figure 5C–F). The group with the early application of the CC16 Ab, showed a significantly increased percentage of CD16^+^Ly6G^+^CD45^+^ viable cells in the BAL, compared to the sham, as well as the control and IgG ALI groups, at 6 hours post-CLP (*p* < 0.05, Figure 5C). After 24 hours, there were no differences among the ALI groups, either with or without Ab application (Figure 5D). Interestingly, in the ALI group with late CC16 Ab application, after 6 hours, no differences among the double-hit groups were observed (Figure 5E). However, after 24 hours, a significantly increased percentage of CD16^+^Ly6G^+^CD45^+^ viable cells in the BAL, compared to the sham as well as the control and IgG ALI groups was observed (*p* < 0.05, Figure 5F).

#### 3.1.5. Histological Examination of Lung Injury

**Figure 6:**
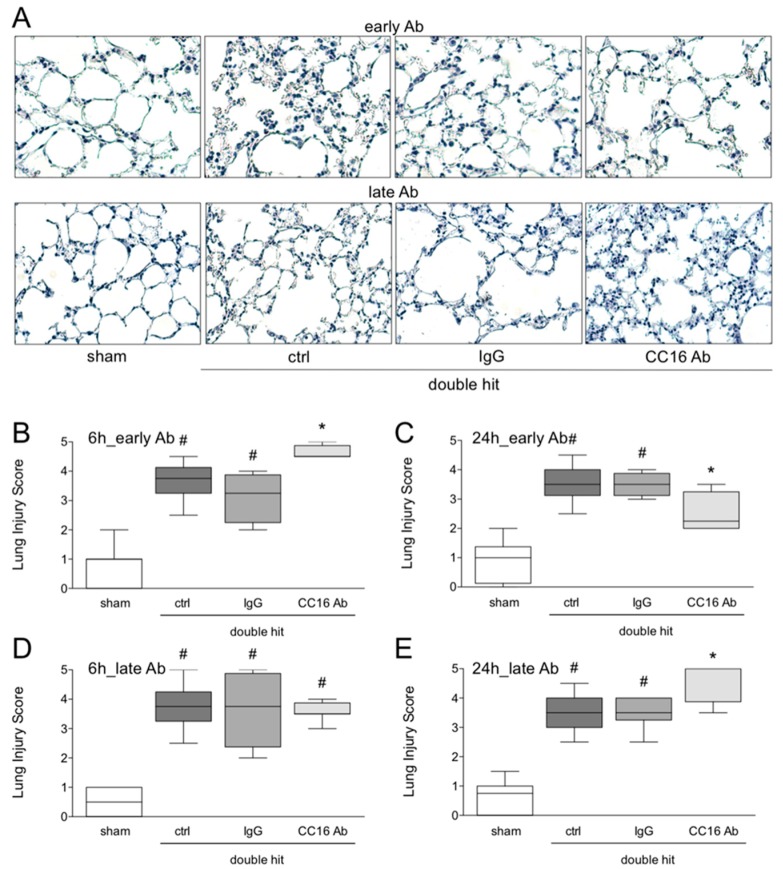
Representative lung sections after hematoxylin-eosin staining are shown (**A**). Lung injury was quantified as lung injury score (**B**–**E**). Interventions with either CC16 antibody (Ab) or IgG control (IgG) antibody were performed either early (post TxT, upper line of (A), and (B,C)) or late (post-CLP, lower line of (A) and (D–E)) to the lungs. Six or 24 hours later, the animals were euthanized for sampling. *: *p* < 0.05 versus all; #: *p* < 0.05 versus sham, *n* = 8.

To analyze whether CC16 modulates the lung injury, histomorphological evaluation of tissue injury after the double hit was assessed. Representative HE-staining is shown in Figure 6A. The lung injury increased significantly in the control group compared to the corresponding sham group, at 6 and 24 hours after CLP (*p* < 0.05, Figure 6B–E). The increased lung damage was significant in the groups that received either the IgG Ab or the CC16 Ab versus sham, at 6 and 24 hours post-CLP (*p* < 0.05, Figure 6B–E). The group with the early application of the CC16 Ab has shown a significantly increased lung injury, as compared to the sham as well as the control and IgG ALI groups, 6 hours post-CLP (*p* < 0.05, Figure 6B). Interestingly, after 24 hours in the same group with the early application of the CC16 Ab, there was a significant decrease in lung injury, compared to the control and IgG acute lung injury groups (*p* < 0.05, Figure 6C). In the ALI group with late CC16 Ab application, no differences among the double-hit groups were observed (Figure 6D), however, after 24 hours, a significantly more lung damage in this group, as compared to the control and IgG ALI groups was detected (*p* < 0.05, Figure 6E).

## 4. Discussion

In our recent study, we confirmed the anti-inflammatory potential of CC16 in sepsis-induced ALI, after blunt chest trauma, observations which have been previously demonstrated in non-traumatic lung injury. Upon an early intratracheal neutralization of CC16, following chest trauma, increased neutrophilic infiltration of the lungs and increased lung injury were observed. However, during the observational period of twenty-four hours, lung damage was significantly reduced by this approach. However, late neutralization of CC16, after CLP, increased neutrophilic infiltration and deteriorated lung injury, after twenty-four hours. Ameliorating early immunosuppression after sepsis-induced ALI after blunt chest trauma, hides the beneficial potential of the early observation period. However, late promotion of inflammation at a slightly later stage of ongoing sepsis-induced ALI was not beneficial to lung injury, within the evaluated time frame of twenty-four hours. The data must be interpreted carefully since a prolonged observation period was not included. Although the anti-inflammatory potential of CC16 in the underlying model has clearly been confirmed, it remains unanswered if the results were caused by the timing of the antibody application or the kinetics of the disease. Thus, although there are some early benefits of promoting inflammation during sepsis-induced ALI development, it is possible that the resolution of lung injury that was observed might just denote a resolution of the inflammatory infiltration, since the animals were not followed longitudinally. In summary, for the short observational period, the anti-inflammatory CC16 might have exerted anti-inflammatory effects in the underlying model; however, its pathological role remains to be further studied. With regard to the quantification of pulmonary CC16 levels upon antibody administration, no significant changes were observed, despite a trend of decrease in the CC16 levels after the double hit. It still remains to be elucidated in further studies if the target itself is modified or not. The data suggest that the antibody might prevent the action of CC16 and not its level, yet in future studies this should be analyzed in further detail.

The development of post-traumatic lung injury depends on multiple factors including the trauma itself, as well as the consecutive release of pathogen-associated molecular patterns and damage-associated molecular patterns (DAMPs) [21,30]. Caused by trauma, the prominent inflammatory response in the lung tissue and airways implies the (intrapulmonary) release of pro-inflammatory chemokines and cytokines, i.e., IL-6 and CXCL5, which promote the chemotaxis and pulmonary neutrophil infiltration, as well as the release of nitrogenous factors [8,10,12]. Since IL-6 is increased systemically and locally in the lungs, our data suggest an ALI-associated pro-inflammatory response. CXCL5 is a neutrophil chemoattractant associated with lung inflammation in mice [31,32]. Moreover, neutralization of pulmonary CXCL5, suppressed neutrophilic inflammation in a mouse model of endotoxin-induced ALI, thereby indicating its prominent role in this pathology [32,33]. Our data showed that increased neutrophilic infiltration after twenty-four hours was paralleled by increased CXCL5 levels in the lungs. However, at six hours, only increased neutrophilic infiltration was observed, suggesting that other factors are involved in neutrophil trafficking. However, CC16 differentially influenced neutrophil chemotaxis, since CXCL5 was highly increased in the ALI group, with early neutralization of CC16, but interestingly, the immigration of neutrophils was not markedly changed twenty-four hours after ALI. CXCL5 has a protective role in atherosclerosis, by directly controlling macrophage-foam cell formation [34]. Thus, significantly increased CXCL5 upon CC16 neutralization, might also be linked to modulations of the migratory behavior of other immune cells.

There are only sparse data describing the CC16 release or its distribution in the lungs. Actually, most research data confirm that CC16 is released from damaged lungs to blood circulation, either through a mechanical injury or, for example endotoxin inhalation [17,18,35]. It is evident that inhalation exposure to lipopolysaccharide induces an intravascular leakage of CC16 [35]. Together with studies from our own group showing that the volume of mechanical lung injury directly correlates with systemic CC16 levels, this confirms a “transition” of CC16 from local to the systemic milieu. Those observations further validate plasma CC16 as a noninvasive tool for the detection of alveolocapillary barrier permeability. However, in an interesting work, the authors found that CC16 was not only present in club cells but also in alveolar epithelial cells [13]. Thus, the authors concluded from their work, that aside from its proposed clinical biomarker characteristic to predict the occurrence of respiratory complications, there is accumulating evidence that CC16 might exert important pathophysiological effects, during the development of lung complications. Our findings might indicate this as well.

Interestingly, the idea of modulating inflammation after traumatic injury in order to prevent infections or organ complications, is challenging due to the high complexity of the inflammatory response to a traumatic injury with numerous mechanisms, which directly or indirectly affects the local pulmonary or systemic inflammation. Nevertheless, in a patient study, the clinical status of severely injured patients with concomitant chest trauma improved significantly, after the use of antioxidant substances [36]. Although the exact underlying pathomechanisms in trauma-induced ALI remain elusive, such studies provide promising and encouraging results regarding the therapeutic strategies. In line with other studies, here, we showed that CC16 exerts anti-inflammatory effects upon blunt chest trauma and sepsis-induced lung inflammation and injury. Recently, Pang et al. showed that recombinant CC16 ameliorated cigarette-smoke-induced lung inflammation and outcome in a murine model of COPD [37]. These results are confirmed by the review of Laucho-Contreras et al. showing that the high expression of CC16 was associated with a reduced inflammation and cellular injury in mice [14]. In patients suffering from pulmonary morbidities such as COPD or Asthma, CC16 is known to play a pivotal role in the pathological course and has a putative protective function during cigarette-smoke exposure [14,38]. Therefore, modulation of inflammation via CC16 might provide a new therapeutic strategy for balancing inflammation. Interestingly, these studies suggest a putative role of bronchial CC16 against smoke-associated lung damage [38]. In our model, the anti-inflammatory effects of CC16 have been confirmed in the CLP-induced ALI upon blunt chest trauma. However, although early “hyperinflammation” by CC16 neutralization has been observed, and been followed by a reduced lung damage sequelae to “hyperinflammation”, the long-term effects are still elusive. Whereas the above-discussed studies indicate ameliorated pulmonary damage by modulating CC16 concentrations in non-traumatic lung injury, our study shows conflictive data with regard to organ damage. It remains questionable why the early inhibition of CC16 improves the pulmonary recovery, following the double hit, while late inhibition further increases lung injury. Yet, as discussed above, this observation might be caused by the timing of the evaluation. Additionally, considering that the timeline and the later protracted inflammation following sepsis, favor hospital-acquired infections and worsens patient’s outcome through immunosuppression [25], the obtained data were intriguing. Upon traumatic insult, as well as throughout medical care or surgical procedures, severely traumatized patients are subjected to endogenous or exogenous damage-associated molecular patterns, which mediate the systemic inflammatory response syndrome (SIRS) or immunosuppression and the clinical consequences in terms of organ failure and infections [25]. Thus, considering the post-traumatic immunosuppression, it seems reasonable that CC16 as an anti-inflammatory mediator might be detrimental to the underlying model. However, with regard to hyperinflammation, the associated SIRS, and the “second hit” hypothesis implying a detrimental hyperinflammatory state upon trauma, CC16, on the other hand, might exert beneficial effects. This question remains unanswered at the moment.

### Limitations

Although we were able to reliably reproduce sepsis-induced ALI in a blunt chest trauma model and show the anti-inflammatory effects of CC16, this study also had some limitations. Keeping in mind the welfare of animals, the number of animals in each group was limited to eight animals. Furthermore, the murine inflammatory pathway differs from human inflammatory cascade. In contrast, all mice were spontaneously breathing and the effects of mechanical ventilation, which is frequently necessary following severe chest trauma, could not be evaluated in this study. With regard to the lung parenchyma analysis, it remains important to mention that the lungs were completely flushed for BAL and that subsequently, those samples were used for further parenchymal analysis. Although all groups underwent the identical procedures, the BAL process could bias the results of parenchymal and histological analysis. Therefore, this critical issue should be omitted in future by doing selective lung BAL (one side only), in order to save the other lung for further analysis. Additionally, other interventional groups, such as isolated TxT, might provide further information on the importance of CC16, during the development of ALI. Another limitation of the study is the unanswered question of whether the antibody prevents the action of CC16 or simply its levels. Although the data allow some speculations that the abrogated action might be the causative factor for the observed results, further studies are necessary to elaborate this in detail. Overall, despite the study dealing with early responses of lung injury and CCL16 inhibition, elaborating long-term impacts of CCL16 inhibition would be interesting as well. As an evident limitation of the present study, this should be evaluated in the future.

## 5. Conclusions

We observed anti-inflammatory effects of CC16, following sepsis-induced ALI after blunt chest trauma in mice. Early local intrapulmonary inhibition of CC16 reduced lung damage after twenty-four hours, indicates a protective effect during the early ALI inflammation. Later inhibition of CC16 resulted in the deteriorated pulmonary outcome. However, this effect might be followed by a reduced lung damage, since delayed antibody application might induce delayed effects as well. The pathological relevance of intrapulmonary CC16 for the development of trauma-related complications should be evaluated further.
